# Iodin as an Astringent, Antiseptic, Disinfectant and Germicide in Mouth Diseases

**Published:** 1910-08-15

**Authors:** Eugene S. Talbot

**Affiliations:** Chicago


### IODIN AS AN ASTRINGENT, ANTISEPTIC, DISINFECTANT AND GERMICIDE IN MOUTH DISEASES.

EUGENE S. TALBOT, M.D., D.D.S., CHICAGO.

For an astringent, antiseptic, disinfectant and germicide in the mouth, there is nothing so convenient and quick acting as tincture of iodin. I have used this drug as an astringent, antiseptic and disinfectant since 1878, but did not know of its germicidal properties until six years later, although I was obtaining the desired results of germ destruction.

Patients present themselves with fetor of the breath pus about the teeth, inflamed gums, diseased alveolar processes, acid mucus and saliva, the latter being also ropy and stringy, plaques on the teeth and decay with all the forms of bacteria from the most harmless to the more dangerous pathogenic microorganisms such as the pneumococcus, diphtheria bacillus, tubercle bacillus and the germs of children's diseases. Miller has demonstrated more than fifty varieties of microorganisms in the mouth. Pus germs are often present in and about the necks of the teeth and easily infect wounds and inflamed tissue. These germs are also taken into the stomach at every swallow and some pass through into the intestines. While most of the better class of patients possess fairly cleanly mouths, yet from 12 to 20 per cent. of all patients have pus germs in the oral cavity.

Tooth decay is due to lactic-acid ferment and nearly every person has it to a greater or less extent. Clinic and dispensary patients, and especially the poorer classes who never use brushes, washes or powders in the mouth, possess regular cesspools of filth. Before the fingers, even, are placed in such mouths, the mucous membrane, gums and teeth should be thoroughly painted—completely covered—with tincture of iodin. Enough iodin should be applied to run in between the gums and the teeth, and then it should be allowed to dry; afterward the mouth should be rinsed with water.

Attention was first called to this method of procedure by my researches on interstitial gingivitis. The pathology of this disease showed the inflammation to be deep-seated throughout the alveolar process. In order to reach this inflammation, iodin alone would penetrate the bone.

All people after the temporary teeth erupt have interstitial gingivitis more or less throughout life. The mouth of every patient of mine, therefore, who has any operation on the mouth or teeth, since I began this method, is treated to an iodin bath before or after the operation. This may occur every day, every other day or whenever the patient has an appointment.

The officinal tincture of iodin contains 7 per cent. of iodin dissolved in alcohol to which is added 5 per cent. of potassium iodid. This preparation, if used often will cause the mucous membrane to become tender and sore; it will also, in some patients, destroy the mucous surface. To overcome this difficulty, many years ago, I formulated the following, which I have called iodoglycerole:

| | |
|---|---|
| Zinc iodid | 15 parts |
| Water | 10 parts |
| Iodin | 25 parts |
| Glycerin | 50 parts |

The proportions were suggested by Prof. C. S. N. Hallberg of the University of Illinois School of Pharmacy, in order to obtain a clear preparation without deposits. As compared with the ordinary tincture of iodin, its astringent properties are greatly increased, the glycerin causes rapid absorption and the irritating effects are reduced to a minimum. The penetrating effect is remarkable. The glycerin thickens the preparation and prevents it from mixing with the saliva and running over the mouth as the ordinary tincture will do. Long, round, wood applicators can be obtained at the drug and instrument houses and on one end cotton is wound; this is saturated with the preparation and the gum margins above and below painted. The jaws are closed, the lips and cheeks distended and the application made as before, the teeth are also covered; the lips and cheeks are held away from the jaws until the iodin has dried.

No one drug will destroy lactic-acid bacilli and their ferment and thus stop tooth decay like iodin. Frequent applications will destroy all germs in the mouth and reduce mouth and general disease to a minimum.

Before operations on the mouth and fauces, the gums, teeth and mucous membrane should be given an application of iodin; especially in the removal of tonsils. After this treatment, and after the iodin has become absorbed, the tonsil or part to be operated on should receive similar treatment. This application should be made from 10 to 15 minutes before operation.

To prevent contagions and infections among public school children, their teeth, gums, and mucous membrane should be painted with iodoglycerole as often as once a month, during the school term. This method will greatly reduce tooth decay.—*A. M. A. Journal.*

